# PReS-FINAL-2022: Juvenile idiopathic arthritis after allogeneic bone marrow transplantation: a case report

**DOI:** 10.1186/1546-0096-11-S2-P35

**Published:** 2013-12-05

**Authors:** E Tronconi, A Miniaci, A Pession

**Affiliations:** 1 Pediatric Unit, University of Bologna, Bologna, Italy

## Introduction

The allogeneic bone marrow transplantation is a therapeutic weapon for treating severe and drug-resistant autoimmune diseases determining the resolution and improvement in the quality of life. On the other hand autoimmune disorders can develop after a hematopoietic stem cell transplantation (HSCT).

## Objectives

AF came to our attention when she was 14 years old for swelling and pain (8 at the Numeric Rating Scale) in the proximal interphalangeal (IFP) joint of the II and III finger of the right hand, painful IFP of the III finger of the left hand accompanied by morning stiffness which lasted for 2 hours and swelling with functional impairment of hands, wrists and knees.

Blood count showed increased inflammatory indexes, antinuclear antibody (ANA) positivity (1:640 in Hep2 cells) and rheumatoid factor negative. The RM of the hands showed endoarticular fluid in both carpal bones and in the radioulnar joint with signs of synovitis; synovitis alone was also present in metacapophalangeal joint of the II, III and IV finger of the left hand and II and III on the right, IFP of III finger left and II and III right, carpometacarpal joint of the I finger bilaterally. This clinical findings were characteristic of Juvenile Idiopathic Arthritis polyarticular rheumatoid factor negative subtype according to the ILAR classification.

The past medical history of our young girl was particularly important. When she was 18 month old, she was diagnosed of acute lymphoblastic leukemia and started chemotherapy according to AEIOP LAL 9502 protocol. One month after the re-induction phase she presented a disease relapse in the bone marrow so she started a chemotherapy cycle of second degree and the research for an unrelated donor because no familiar HLA-matched donor was present. When she was 3 years old, she underwent hematopoietic stem cells transplantation of peripheral-derived source. Ten months later, during the tapering of immunosuppressive therapy, she presented skin lesions resembling chronic GVHD so she started again immunosuppressive therapy with tacrolimus and mycophenolate mofetil. Later she developed restrictive respiratory syndrome, ovarian insufficiency in replacement therapy and a thyroiditis ultrasonography aspect without hormonal alterations.

This important personal history challenged us in the choice of therapy. She started with NSAID (Naproxen) with only partial relief. For the severe polyarticular involvement, after the discussion with the hematologists, we decided to introduce a steroid agent. The girl had a good response but, during steroid tapering, pain and functional impairment of legs and arms reappeared with morning stiffness and swelling of hands. For this reason we finally decided to start Methotrexate.

## Conclusion

We don't know exactly if juvenile idiopathic arthritis in our patient is a de novo autoimmune disease induced by the transfer of a heterologous immunological system or a rare presentation of graft-versus-host-disease, however MTX therapy was effective and improved the symptomatology with a complete remission after 2 months.

## Disclosure of interest

None declared.

